# *De novo* likelihood-based measures for comparing genome assemblies

**DOI:** 10.1186/1756-0500-6-334

**Published:** 2013-08-22

**Authors:** Mohammadreza Ghodsi, Christopher M Hill, Irina Astrovskaya, Henry Lin, Dan D Sommer, Sergey Koren, Mihai Pop

**Affiliations:** 1 Center for Bioinformatics and Computational Biology, University of Maryland, College Park, Maryland, USA; 2 The Genome Center, Columbia University Medical Center, New York, New York, USA; 3 National Biodefense Analysis and Countermeasures Center, Battelle National Biodefense Institute, Frederick, Maryland, USA; 4 3120F Biomolecular Sciences Building, University of Maryland, College Park, Maryland, USA

## Abstract

**Background:**

The current revolution in genomics has been made possible by software tools called genome assemblers, which stitch together DNA fragments “read” by sequencing machines into complete or nearly complete genome sequences. Despite decades of research in this field and the development of dozens of genome assemblers, assessing and comparing the quality of assembled genome sequences still relies on the availability of independently determined standards, such as manually curated genome sequences, or independently produced mapping data. These “gold standards” can be expensive to produce and may only cover a small fraction of the genome, which limits their applicability to newly generated genome sequences. Here we introduce a *de novo* probabilistic measure of assembly quality which allows for an objective comparison of multiple assemblies generated from the same set of reads. We define the quality of a sequence produced by an assembler as the conditional probability of observing the sequenced reads from the assembled sequence. A key property of our metric is that the true genome sequence maximizes the score, unlike other commonly used metrics.

**Results:**

We demonstrate that our *de novo* score can be computed quickly and accurately in a practical setting even for large datasets, by estimating the score from a relatively small sample of the reads. To demonstrate the benefits of our score, we measure the quality of the assemblies generated in the GAGE and Assemblathon 1 assembly “bake-offs” with our metric. Even without knowledge of the true reference sequence, our *de novo* metric closely matches the reference-based evaluation metrics used in the studies and outperforms other *de novo* metrics traditionally used to measure assembly quality (such as N50). Finally, we highlight the application of our score to optimize assembly parameters used in genome assemblers, which enables better assemblies to be produced, even without prior knowledge of the genome being assembled.

**Conclusion:**

Likelihood-based measures, such as ours proposed here, will become the new standard for *de novo* assembly evaluation.

## Background

The genome sequence of an organism is a critical resource for biologists trying to understand the organism’s function and evolution. Obtaining this sequence is difficult as modern sequencing technologies can only “read” small pieces of the genome (called *reads*). The fact that these tiny *reads* (under a few thousands of basepairs in length) can be glued together to reconstruct genomes comprising millions to billions of basepairs is by no means evident and was the subject of vigorous scientific debate during the early days of sequencing technologies [1,2]. The modern genomic revolution was in no small part made possible by the development of algorithms and computational tools called *genome assemblers* able to reconstruct near-complete representations of a genome’s sequence from the fragmented data generated by sequencing instruments. Despite tremendous advances made over the past 30 years in both sequencing technologies and assembly algorithms, genome assembly remains a highly difficult computational problem. In all but the simplest cases, genome assemblers cannot fully and correctly reconstruct an organism’s genome. Instead, the output of an assembler consists of a set of contiguous sequence fragments (*contigs*), which can be further ordered and oriented into *scaffolds*, representing the relative placement of the contigs, with possible intervening gaps, along the genome.

Theoretical analyses of the assembly problem, commonly formulated as an optimization problem within an appropriately defined graph, have shown that assembly is NP-hard [3,4], i.e., finding the correct optimal solution may require an exhaustive search of an exponential number of possible solutions. The difficulty of genome assembly is due to the presence of repeated DNA segments (*repeats*) in most genomes. Repeats longer than the length of the sequenced reads lead to ambiguity in the reconstruction of the genome – many different genomes can be built from the same set of reads [5,6].

As a result, practical implementations of assembly algorithms (such as ABySS [7], Velvet [8], SOAPdenovo [9], etc.) return just an approximate solution that either contains errors, or is fragmented, or both. Ideally, in a genomic experiment, assembly would be followed by the scrupulous manual curation of the assembled sequence to correct the hundreds to thousands of errors [10], and fill in the gaps between the assembled contigs [11]. Despite the value of fully completed and verified genome sequences [12], the substantial effort and associated cost necessary to conduct a finishing experiment to its conclusion can only be justified for a few high-priority genomes (such as reference strains or model organisms). The majority of the genomes sequenced today are automatically reconstructed in a “draft” state. Despite the fact that valuable biological conclusions can be derived from draft sequences [13], these genomes are of uncertain quality [14], possibly impacting the conclusions of analyses and experiments that rely on their primary sequence.

Assessing the quality of the sequence output by an assembler is, thus, of critical importance, not just to inform downstream analyses, but also to allow researchers to choose from among a rapidly increasing collection of genome assemblers. Despite apparent incremental improvements in the performance of genome assemblers, none of the software tools available today outperforms the rest in all assembly tasks. As highlighted by recent assembly bake-offs [15,16], different assemblers “win the race” depending on the specific characteristics of the sequencing data, the structure of the genome being assembled, or the specific needs of the downstream analysis process. Furthermore, these recent competitions have highlighted the inherent difficulty of assessing the quality of an assembly. More specifically, all assemblers attempt to find a trade-off between contiguity (the size of the contigs generated) and accuracy of the resulting sequence. Evaluating this trade-off is difficult even when a gold standard is available, e.g., when re-assembling a genome with known sequence. In most practical settings, a reference genome sequence is not available, and the validation process must rely on other sources of information, such as independently derived data from mapping experiments [17], or from transcriptome sequencing [18]. Such data are, however, often not generated due to their high cost relative to the rapidly decreasing costs of sequencing. Most commonly, validation relies on *de novo* approaches based on the sequencing data alone, which include global “sanity checks” (such as gene density, expected to be high in bacterial genomes, measured, for example, through the fraction of the assembled sequence that can be recognized by PFAM profiles [19]) and internal consistency measures [20] that evaluate the placement of reads and mate-pairs along the assembled sequence.

The validation approaches outlined above can highlight a number of inconsistencies or errors in the assembled sequence, information valuable as a guide for further validation and refinement experiments, but difficult to use in a comparative setting where the goal is to compare the quality of multiple assemblies of a same dataset. For example, even if a reference genome sequence is available, while all differences between the reassembled genome and the reference are, at some level, assembly mistakes, it is unclear whether one should weigh single nucleotide differences and short indels as much as larger structural errors (e.g., translocation or large scale copy-number changes) [15] when comparing different assemblies. Furthermore, while recent advances in visualization techniques, such as the FRCurve of Narzisi et al. [21,22], have made it easier for scientists to appropriately visualize the overall tradeoff between assembly contiguity and correctness, there exist no established approaches that allow one to appropriately weigh the relative importance of the multitude of assembly quality measures, many of which provide redundant information [22].

Here we propose an objective and holistic approach for evaluating and comparing the quality of assemblies derived from a same dataset. Our approach defines the quality of an assembly as the likelihood that the observed reads are generated from the given assembly, a value which can be accurately estimated by appropriately modeling the sequencing process. This basic idea was formulated in the 1990’s in the pioneering work of Gene Myers [3], where he suggested the correct assembly of a set of reads must be consistent (in terms of the Kolmogorov-Smirnoff test statistic) with the statistical characteristics of the data generation process. The same basic idea was further used in the arrival-rate statistic (A-statistic) in Celera assembler [23] to identify collapsed repeats, and as an objective function in quasi-species (ShoRAH [24], ViSpA [25]), metagenomic (Genovo [19]), general-purpose assemblers [26], and recent assembly evaluation frameworks (ALE [27], CGAL [28]).

In our paper, we will describe in detail a mathematical model of the sequencing process that takes into account sequencing errors and mate-pair information, and show how this model can be computed in practice. We will also show that this *de novo* probabilistic framework is able to automatically and accurately reproduce the reference-based ranking of assembly tools produced by the Assemblathon [15] and GAGE [16] competitions. Our work is similar in spirit to the recently published ALE [27] and CGAL [28]; however, we provide here several extensions of practical importance. First, we propose and evaluate a sampling-based protocol for computing the assembly score which allows the rapid approximation of assembly quality, enabling the application of our methods to large datasets. Second, we evaluate the effect of unassembled reads and contaminant DNA on the relative ranking of assemblies according to the likelihood score. Finally, we will demonstrate the use of our probabilistic quality measure as an objective function in optimizing the parameters of assembly programs. The software implementing our approach is made available, open-source and free of charge, at: http://assembly-eval.sourceforge.net/

## Methods

### Theoretical foundation for probabilistic evaluation

In this section, we formalize the probabilistic formulation of assembly quality and the model of the sequencing process that allows us to compute the likelihood of any particular assembly of a set of reads. We will show that the proposed probabilistic score is correct in the sense that the score is maximized by the true genome sequence.

#### ***Likelihood of an assembly***

Let *A* denote the event that a given assembly is the true genome sequence, and let *R* denote the event of observing a given set of reads. In the following, we will use the same symbol to denote the assembly sequence and the event of observing the assembly. We will also use the same symbol to denote the set of reads and the event of observing the set of reads.

According to Bayes’ rule, given the observed set of reads, the probability of the assembly can be written as:

(1) $$\mathrm{Pr}[\;A|R]=\frac{\mathrm{Pr}[\;R|A]\mathrm{Pr}[\;A]}{\mathrm{Pr}[\;R]}$$

where Pr[ *A*] is the *prior probability* of observing the genome sequence *A*. Any prior knowledge about the genome being assembled (e.g., approximate length, presence of certain genes, etc.) can be included in Pr[ *A*]; however, for the purpose of this paper, we will assume that this prior probability is constant across the set of “reasonable” assemblies of a same set of reads. Given commonly available information about the genomes, formulating a precise mathematical framework for defining Pr[ *A*] is an extensive endeavor beyond the scope of this paper.

Similarly, Pr[ *R*] is the prior probability of observing the set of reads *R*. Since our primary goal is to compare multiple assemblies of a same set of reads, rather than to obtain a universally accurate measure of assembly quality, we can assume Pr[ *R*] is a constant as well. Thus, for the purpose of comparing assemblies, the values Pr[ *A*|*R*] and Pr[ *R*|*A*] are equivalent. The latter, the posterior probability of a set of reads, given a particular assembly of the data, can be easily computed on the basis of an appropriately defined model of the sequencing process and will be used in our paper as a proxy for assembly quality.

Under the assumption that individual reads are independent of each other (violations of this assumptions in the case of mate-pair experiments will be discussed later in this section), $\mathrm{Pr}[\;R|A]=\underset{r\in R}{\prod }\mathrm{Pr}[\;r|A]$. If the set of reads is unordered, we need to account for the different permutations that generate the same set of reads. As this value is a constant for any given set of reads, we ignore it in the rest of our paper.

Pr[ *R*|*A*], hereafter referred to as *p*_*r*_, can be computed using an appropriate model for the sequencing process. Throughout the remainder of the paper, we will discuss increasingly complex models and their impact on the accuracy of the likelihood score.

#### ***True genome obtains the maximum likelihood***

Any useful assembly quality metric must achieve its maximum value when evaluating the true genome sequence; otherwise, incorrect assemblies of the data would be preferred. We prove below that the likelihood measure proposed in our paper satisfies this property.

Assuming that we have a set of reads *R* from the true genome, produced by generating exactly one single-end read from each location in the genome without errors and with a fixed length. Given the set of reads *R*, the probability a particular read is generated from the true genome is precisely the number of times the read occurs in *R* divided by the size of *R* (note that multiple reads can have the same sequence, e.g., when generated from repeats). Let *N*_*s*_ denote number of times that the sequence *s* occurs in *R*, and *q*_*s*_ = *N*_*s*_/|*R*| denote the probability that sequence *s* is generated from the true genome. To show that the true genome maximizes the likelihood score, let us assume that we have some assembly *A* and *p*_*s*_ is the probability that the sequence *s* is generated from the assembly *A*.

Given assembly *A*, our likelihood score is then the product of ${p_{s}}^{N_{s}}$ over all sequences *s* in *S*, which can be rewritten as $\underset{s\in S}{\prod }{p_{s}}^{q_{s}|R|}={\left(\underset{s\in S}{\prod }{p_{s}}^{q_{s}}\right)}^{\left|R\right|}$. Now, note that since |*R*| is a fixed constant, maximizing the likelihood score is equivalent to maximizing

$$\begin{array}{ll} \underset{s\in S}{\prod }{p_{s}}^{q_{s}} & \end{array}$$

The likelihood can be re-written as

$$\begin{array}{lll} \text{log}(\underset{s\in S}{\prod }{p_{s}}^{q_{s}}) & =\underset{s\in S}{\sum }q_{s}\text{log}p_{s}\; & \\ & =\underset{s\in S}{\sum }q_{s}\text{log}(\frac{p_{s}}{q_{s}})+\underset{s\in S}{\sum }q_{s}\text{log}q_{s}\; & \\ & =-D_{\text{KL}}(Q||P)-H(Q),\; & \end{array}$$

where *D*_*KL*_(*Q*||*P*) is the KL-divergence for the distributions *Q* and *P*, and *H*(*Q*) is the Shannon entropy of *Q*. Since the KL-divergence is always non-negative and only equal to 0 if and only if *Q* = *P*, the average probability is maximized if the assembly is equal to the true genome.

Even though the true genome does maximize the likelihood in this model, there may be other assemblies that achieve the same optimal score as long as these assemblies yield probabilities *p*_*s*_ which are equal to the probabilities *q*_*s*_ for every sequence *s*. This can happen, for example, in the case of a misassembly that is nonetheless consistent with the generated reads. This situation highlights the loss of information inherent in modern sequencing experiments – without additional long-range information, the information provided by the reads themselves is insufficient to distinguish between multiple possible reconstructions of a genome [6].

#### ***Error-free model for fragment sequencing***

The most basic model for the sequencing process is the *error-free model*. In this model, we assume reads of a given fixed length (a more general read length distribution can be included in the model but would not impact comparative analyses of assemblies derived from a same set of reads). We further assume that reads are uniformly sampled across the genome, i.e., that every position of the genome is equally likely to be a starting point for a read. This simplifying assumption is made by virtually all other theoretical models of genome assembly, despite the biases inherent to all modern sequencing technologies. A more accurate, technology-dependent, model can be obtained by including additional factors that account, for example, for DNA composition biases. For the purpose of generality, we restrict our discussion to the uniform sampling model. Furthermore, for the sake of simplicity, we assume (1) that the true genome consists of a single circular contiguous sequence, (2) that our assembly is also a single contig, and (3) that every read can be mapped to the assembly. We will later discuss extensions of our model that relax these assumptions.

Under these assumptions, we can compute the probability of a read *r* given the assembled sequence as:

(2) $$p_{r}=\frac{n_{r}}{2L}$$

where *n*_*r*_ represents the number of places where the read occurs in the assembled sequence of length *L*. The factor 2 is due to the fact that reads are sampled with equal likelihood from both the forward and reverse strands of a DNA molecule. This formulation was previously used by Medvedev *et al.*[26] to define an objective function for genome assembly.

### ***A realistic model of the sequencing process***

The error-free model outlined above makes many simplifying assumptions that are not representative of real datasets. Here we demonstrate how the model can be extended to account for artifacts such as sequencing errors, mate-pair information, assemblies consisting of multiple contigs, and the presence of un-mappable reads.

#### ***Sequencing errors***

All current technologies for sequencing DNA have a small but significant probability of error. Here we focus on three common types of errors: the insertion, deletion, and substitution of a nucleotide.

In the error-free model, the probability of a read having been generated from a position *j* in the sequence is one if the read exactly matches the reference at that position and zero otherwise. We now extend this model such that the probability of each read having been generated from any position *j* of the reference is a real value between zero and one, representing the likelihood that a sequencing instrument would have generated that specific read from that specific position of the reference. This value clearly depends on the number of differences between the sequence of the read and the sequence of the reference at position *j*. Given the assembled sequence, the probability of a particular read will be the cumulative probability of the read across all possible locations in the genome.

Specifically, let us denote the probability that read *r* is observed by sequencing the reference, *ending* at position *j* by *p*_*r*,*j*_. Then, the total probability of the read *r* is

(3) $$\begin{array}{ll} p_{r}=\frac{\overset{L}{\underset{j=1}{\sum }}p_{r,j}^{\text{forward}}+\overset{L}{\underset{j=1}{\sum }}p_{r,j}^{\text{reverse}}}{2L} & \end{array}$$

The individual probabilities *p*_*r*,*j*_ can be computed if we do not model insertion and deletion errors and only allow substitution errors which occur with probability *ε*. The per-base probability of a substitution error can be set individually for each based on the quality value produced by the sequencing instrument. Then, *p*_*r*,*j*_ = *ε*^*s*^(1 - *ε*)^*l*-*s*^, where *s* is the number of substitutions needed to match read *r* to position *j* of the reference sequence. In the more general case, *p*_*r*,*j*_ values can be computed using dynamic programming.

#### ***Exact probability calculation via dynamic programming***

For a model of the sequencing process that allows insertions, deletions, and substitutions with specific probabilities, we can exactly compute probability, *p*_*r*_ = Pr[ *r*|*A*], of observing a read *r* given an assembly *A* using a dynamic programming algorithm. In general, we want to find the sum of the probabilities of all possible alignments of a read to a position of the assembly.

The number of such possible alignments grows exponentially as a function of read length. Most of those alignments have a very small probability. However, several alignments may have probabilities that are equal or close to the optimal. For example, when aligning the sequence **ACG** to the assembly **ACCG**, both **A-CG** and **AC-G** are optimal alignments and have the same probability. The contribution of all such alignments must be taken into account when computing the probability for a read.

We use a dynamic programming algorithm (similar to the “forward” algorithm in Hidden Markov Models) to efficiently calculate the sum of the probabilities of all alignments of a read to the assembly as follows. In the formula (3), $p_{r,j}^{\text{forward}}$ and $p_{r,j}^{\text{reverse}}$ are the sum of the probabilities of *all* possible alignments of the read *r* to, respectively, the reference and its reverse complement, ending at position *j*.

We define *T*[ *x*,*y*] as the probability of observing prefix [ 1…*y*] of the read *r*, if *y* bases are sequenced from the reference, ending at position *x*. Therefore, *p*_*r*,*j*_ = *T*[ *j*,*l*]. *T*[ *x*,0] represents the probability of observing an empty sequence if we sequence zero bases and is set to 1. *T*[ 0,*y*] represents the probability of observing prefix [ 1…*y*] of the read if *y* bases are sequenced from the reference, ending at position 0 (before the beginning), and is set to 0.

For *x* ≥ 1 and *y* ≥ 1, *T*[ *x*,*y*] is recursively defined:

(4) $$\begin{array}{ll} T[\;x,y]\;= & T\;[x-1,y-1]\mathrm{Pr}[\;\text{Substitute}(A[\;x],r[y]\;)]\\ & +T[\;x,y-1]\mathrm{Pr}[\;\text{Insert}(r[y]\;)]\\ & +T[\;x-1,y]\mathrm{Pr}[\;\text{Delete}(A[\;x]\;)], \end{array}$$

where *r*[*y*] and *A*[ *x*] represent the nucleotides at positions *y* and *x* of the read *r* and the assembly *A*, respectively. Pr[ Substitute(*A*[ *x*],*r*[*y*])] is the probability of observing the nucleotide *r*[*y*] by sequencing the nucleotide *A*[ *x*]. In our experiments, we did not distinguish between different types of errors and considered their probability to be *ε* and the probability of observing the correct nucleotide to be 1-*ε*.

The dynamic programming algorithm outlined above has a running time of *O*(*lL*) per read. Even though the running time is polynomial, it is slow in practice. However, we can speed it up by using alignment seeds. The seeds would give us the regions of the assembly where a read may align with high probability. We can apply the dynamic programming only to those regions and get a very good approximate value of the total probability. We use exact seeds (*k*-mers) of a given length to build a hash index of the assembly sequence. Then, each read is compared to the regions where it has a common *k*-mer with the assembly sequence.

#### ***Mate pairs***

Many of the current sequencing technologies produce paired reads – reads generated from the opposite ends of the same DNA fragment. This information is extremely valuable in resolving genomic repeats and in ordering the contigs along long-range scaffolds; however, the paired reads violate the assumption that reads are sampled independently, that we made in the discussion above. To address this issue, we can use the pairs rather than the individual reads as the underlying objects from which the assembly likelihood is computed. To address the possibility that assembly errors may result in violations of the constraints imposed by the paired reads, we only consider pairs for which both ends align to a same contig or scaffold within the constraints imposed by the parameters of the sequencing experiment. Any pairs that violate these constraints get classified as unassembled. Note that in addition to sequencing errors, we now also handle fragment sizing errors – deviations of the estimated distance between paired reads from the distance implied by the sequencing experiment. We model the distribution of fragment sizes within a same library by a normal distribution, using user-supplied parameters, and use this information to appropriately scale the likelihood estimate for each possible placement of a mate pair along the genome.

We modify the dynamic programming recurrence from formula (4) to handle the probability calculation for the paired reads as follows. The probability of the first read in the pair is calculated as the same as in the formula (4). For the second read, we adjust the dynamic programming to ensure that it is aligned within a certain distance downstream of the alignment of the first read. We modify the first column of the dynamic programming table of the *second* read in the pair to take into account the distance from the first read.

Formally, given a paired read, we define *T*_2_[ *x*,*y*] as the probability of observing prefix [ 1…*y*] of the 2nd read in the pair, if *y* bases are sequenced from the reference, ending at position *x*.

Assume that the second read occurs after the first read and is separated by a normally-distributed distance with mean *μ* and with a standard deviation *σ*.

Therefore,

(5) $$\begin{array}{ll} \;T_{2}[\;x,0]=\overset{x}{\underset{i=1}{\sum }}\mathrm{Pr}[\;\text{insert}(x-i)|N(\mu ,\sigma )))]+T_{1}[\;x-i,l], & \end{array}$$

where Pr[ insert(*n*)|*N*(*μ*,*σ*)))] is the probability of observing an insert size of length *n* from a normal distribution with parameters *μ* and *σ*, and *l* is the length of the first read in the pair.

Instead of using two tables, we can concatenate the read pair together with a special character (*M*), which will signal when the insert size should be taken into account.

For *x* ≥ 1 and *y* ≥ 1, *T*[ *x*,*y*] is recursively defined as follows:

(6) $$\begin{array}{ll} \;T[\;x,y] & =\text{if}r[y]=\\ & =\;M\left\{\begin{array}{l} \overset{x}{\underset{i=1}{\sum }}\mathrm{Pr}[\;\text{insert}(x-i)|N(\mu ,\sigma )))]\;+T[\;x-i,y-1] \end{array}\right)\\ & \;\text{else}\left\{\begin{array}{l} T[\;x-1,y-1]\mathrm{Pr}[\;\text{Substitute}(A[\;x],r[y]\;)]\\ \;+T[\;x,y-1]\mathrm{Pr}[\;\text{Insert}(r[y]\;)]\\ \;+T[\;x-1,y]\mathrm{Pr}[\;\text{Delete}(A[\;x]\;)] \end{array}\right) \end{array}$$

#### ***Assemblies containing more than one contig***

As we mentioned in the introduction, the output of an assembler usually consists of a (large) set of contigs rather than one single contig, representing the genome being assembled. In the extreme case, an “assembler” may return the set of unassembled input reads (or the set of all k-mers in De Bruijn-based assemblers) as its output. Our likelihood score must be modified to account for such fragmented assemblies.

In practice, most assemblers join contigs only if they overlap by more than a certain number of bases; however, we only consider the case where contigs are non-overlapping substrings of the true genome. In this case, the length of the original genome must be *at least* the sum of the lengths of the contigs, that is, $\sum L_{j}$, where *L*_*j*_ is the length of the *j*th contig. Therefore, the probability of every read is at most:

(7) $$\begin{array}{l} \frac{n_{r}}{2\sum L_{j}} \end{array}$$

Overlapping contigs can be handled by reducing the length of the contigs by a value representing the minimum overlap required by the assembler, as performed, for example, in Genovo [19].

#### ***Reads that do not align well***

In practice, popular assemblers do not incorporate every read in the assembly. Possible reasons include assembly errors (such as collapsed tandem repeats), reads with high error rates, or contamination in the DNA sample. These “singleton” or “chaff” reads cannot be modeled by our likelihood approach as the likelihood of any assembly that does not incorporate every read is zero. When sequencing errors are modeled, every read obtains a non-zero likelihood, even if it does not align to the assembly. Since, in general, a non-trivial fraction of the total set of the reads cannot be mapped to the assembly, by their sheer number, the singleton reads would dominate the probability calculation.

To account for this factor, we argue that for any read that does not align well, the overall probability of the assembly should not be lower than the probability of the same assembly when the missing read is appended to its sequence as a separate contig. The effect of such an addition on the overall probability can be calculated as follows. First, the probability of observing this read exactly, $\left(\frac{\mathrm{Pr}[\;\text{exact match}]}{2L}\right)$, is multiplied to the product of the probabilities of all mapped reads. Second, the probabilities of the mapped reads are decreased slightly due to the increase in the length of the assembled sequence.

For simplicity, let us assume an error-free model where each read maps to exactly one position on the assembled sequence. Let *k* denote the number of the original reads. The ratio between the new probability for all original reads divided by their probability before adding the new read is:

$$\frac{\frac{1}{{\left(L+l\right)}^{k}}}{\frac{1}{L^{k}}}={\left(\frac{L}{L+l}\right)}^{k}={\left(1-\frac{l}{L+l}\right)}^{k}\approx e^{-\frac{\text{lk}}{L}}$$

Therefore, if the probability of observing a read is less than

(8) $$\frac{\mathrm{Pr}[\;\;\text{exact match}]}{2L}e^{-\frac{l\left|R\right|}{L}},$$

we consider this read as “unmapped” and use formula (8) as its probability. The probability of an exact match Pr[ exact match] is approximated by (1-*ε*)^*l*^, where *ε* is the probability of an error (a mismatch, an insertion, or a deletion).

### Performance considerations

#### ***Estimating the average read likelihood by sampling***

Depending on the specific characteristics of the chosen sequencing model, the computation of the probability Pr[ *R*|*A*] can be expensive for the dataset sizes commonly encountered in current projects (tens to hundreds of millions of reads). In such cases, we can approximate the likelihood of an assembly by using a random subset of the reads *R*^′^ ⊆ *R*. To counteract the effect of the size of the sample on the computed probability, we define the assembly quality as the geometric mean of the individual read probabilities:

(9) $$\text{AP}(R^{\prime })={\left(\underset{r\in R^{\prime }}{\prod }p_{r}\right)}^{\frac{1}{\left|R^{\prime }\right|}}$$

The logarithm of this value (Log Average Probability (LAP)) is reported in the remainder of the paper as the assembly quality “score”:

(10) $$\text{LAP}(R^{\prime })=\underset{10}{\text{log}}\left(\text{AP}(R^{\prime })\right)=\frac{\underset{r\in R^{\prime }}{\sum }\underset{10}{\text{log}}p_{r}}{\left|R^{\prime }\right|}$$

In other words, we define the assembly quality as the average log likelihood of the reads given an assembly. This formulation also allows us to estimate the accuracy of the approximate likelihood value produced by sub-sampling the set of reads. According to sampling theory, the distribution of the scores across multiple samples has the mean equal to the true likelihood of the assembly (computed from all the reads) and a standard error proportional to $\frac{1}{\sqrt{\left|R^{\prime }\right|}}$, i.e., the larger the sample is, the more accurate our estimation is for the likelihood of the true assembly. Since the probability of a read is bounded by formula (8), the variance of the sample can also be bounded by this value.

In practice, we increase the sample size until the assemblies can be unambiguously distinguished by the LAP value. Specifically, we increase the sample size, by binary search, until the LAP values are separated by at least a single standard deviation. The level of subsampling required will, thus, be dependent on the extent of the differences between the assemblies — for very different assemblies, low levels of subsampling are sufficient.

#### ***Approximating the likelihood value using an aligner***

Alternatively, when it is impractical to calculate exact probabilities for large sets of reads, we can approximate these probabilities using fast and memory-efficient alignment search programs, which internally model the sequencing process. We use Bowtie 2 [29] to align the reads to the assembly. However, our programs are easy to adapt for any read alignment tool that stores the alignment results in SAM [30] format.

For each reported alignment, we use the number of substitutions *s* to compute the probability *p*_*r*_. The probability of this alignment, *p*_*r*,*j*_, can be approximated by *ε*^*s*^(1 - *ε*)^*l*-*s*^ and

(11) $$p_{r}=\frac{\underset{j\in S_{r}}{\sum }p_{r,j}}{2L},$$

where *S*_*r*_ is the set of alignments in the SAM file for the read *r*.

We can further extend this equation to mated reads. A pair of mated reads aligns if the distance and orientation of the alignment of the pair are consistent with the experimental design parameters. Given read *i*_1_ and its mate *i*_2_, we compute $p_{\left(i_{1},i_{2}\right)}$ by multiplying the probabilities of individually aligning each mate at their respective positions with the probability that they are separated by their distance from each other. That is,

(12) $$\;p_{\left(i_{1},i_{2}\right)}=\frac{\underset{(j_{1},j_{2})\in S_{\left(i_{1},i_{2}\right)}}{\sum }p_{i_{1},j_{1}}p_{i_{2},j_{2}}\mathrm{Pr}[\;\text{insert}(j_{2}-(j_{1}+l_{1}))]}{2(L-l)},$$

where $p_{i_{1},j_{1}}=\varepsilon^{s_{1}}{\left(1-\varepsilon \right)}^{l_{1}-s_{1}}$. Mate pair insert sizes follow a normal distribution with mean and standard deviation being estimated from the parameters of the sequencing process. Unless otherwise stated, the standard deviation is 10% of the insert size. If only one of the mates, *i*_1_ or *i*_2_, maps, the probability $p_{\left(i_{1},i_{2}\right)}$ is 0. We use (8) to set the probability for this case.

In our experiments, Bowtie 2 was used to approximate the read probabilities for the larger datasets; however, it could be substituted with any other aligner.

### Data sets

The read data for *Rhodobacter sphaeroides* 2.4.1 was downloaded from http://gage.cbcb.umd.edu/data/Rhodobacter_sphaeroides, and the corresponding reference sequence was obtained from the NCBI RefSeq database (NC_007493.1, NC_007494.1, NC_009007.1, NC_007488.1, NC_007489.1, NC_007490.1, NC_009008.1). In addition, two more *Rhodobacter* genomes were selected as reference genomes, specifically *R. sphaeroides* ATCC 17025 (NCBI IDs NC_009428.1, NC_009429.1, NC_009430.1, NC_009431.1, NC_009432.1), and *R. capsulatus* SB1003 (NC_014034.1, NC_014035.1).

The read data for *Stapylococcus aureus* USA300 was downloaded from http://http://gage.cbcb.umd.edu/data/{Staphylococcus}_aureus, and the corresponding reference sequence was obtained from the NCBI RefSeq database (NC_010063.1, NC_010079.1, NC_012417.1). In addition, two more *Stapylococcus* genomes were selected as reference genomes, specifically *S. aureus* 04-02981 (CP001844), and *S. epidermidis* ATCC 12228 (AE015929, AE015930, AE015931, AE015932, AE015933, AE015934, AE015935).

The read data for human chromosome 14 was downloaded from http://gage.cbcb.umd.edu/data/{Hg}_chr14/, and the corresponding reference sequence was obtained from the NCBI RefSeq database (NC_000014.8).

The Assemblathon 1 competition evaluates assemblies on the simulated short read dataset generated from the simulated 110 Mbp diploid genome. The competition provides sequence libraries with varying insert sizes (200-10,000 bp) and coverage (20-40x). Assemblathon 1 allowed teams to submit multiple entries, but for our analyses, we only examine the top ranking assemblies from each team. The raw reads and the consensus sequence of the top ranking assemblies were downloaded from http://korflab.ucdavis.edu/Datasets/Assemblathon/Assemblathon1/.

Also used in our analyses is the *E. coli* K12 MG1655 dataset, generated using Illumina MiSeq technology (300 bp insert size, 370 × coverage) (http://www.illumina.com/systems/miseq/{scientific}_data.ilmn).

For detailed software usage, please see Additional file 1.

## Results

### Performance-related approximations do not significantly affect the likelihood score

The full and exact computation of the assembly likelihood score is computationally intensive and ultimately impractical for the analysis of large genomes sequenced with the next generation technologies. We have highlighted in the Methods section several approaches that can be used to reduce the computational requirements and allow the application of our methods in practical settings, including the computation of the likelihood score on the subsets of the original set of reads and the approximation of the score from the output of an alignment program. As we will show below, our approximations do not affect the comparative ranking of the multiple assemblies derived from a same dataset.

#### ***The likelihood score is robust under sampling***

To assess the effect of subsampling, we relied on a collection of the assemblies of the human chromosome 14 made available by the GAGE assembly ‘bake-off’. We sampled random subsets of increasing size (one trial per size) from the over 60 million reads and computed the likelihood score based only on the sampled reads.

**Figure 1:**
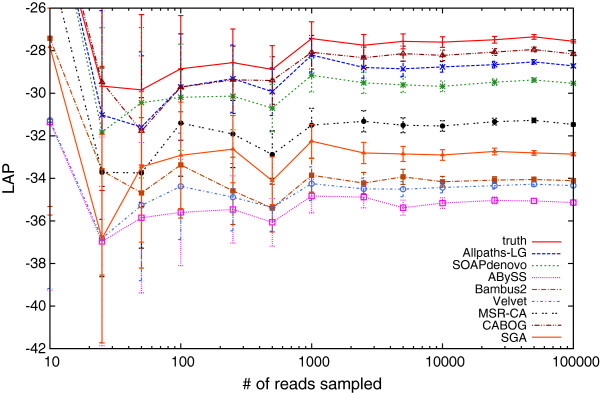
**LAP-based evaluation of the assemblies for the human chromosome 14 via sampling.** The *x*-axis represents the number of sampled reads. For each assembly, we plot the corresponding LAP on a chosen subsample along with the standard deviation. The relative ranking of assemblies becomes fixed with 10,000 reads, which is less than 0.02% of the original reads.

As seen in Figure 1, the overall ranking of the different assemblies stabilizes after sampling just 10,000 reads, i.e., less than 0.02% of the entire dataset. After this point, the scores of individual assemblies differ by more than the standard deviation of the sub-sampled scores, indicating the relative ranking of the assemblies can be determined with high statistical confidence. This result suggests a practical strategy for computing the assembly likelihood wherein datasets of increasing size are repeatedly sampled from the set of reads until the likelihood scores of the compared assemblies can be distinguished from each other. The search for the appropriate sample size can start from a reasonable ‘guess’ (e.g., 0.05% of the total set of reads), which is then iteratively doubled until the likelihood scores are separated from each other by a given multiple of the sampling-induced standard deviation.

#### ***Aligner-based approximation correlates with the dynamic-programming computation of the likelihood score***

As outlined in the Methods section, we relied on an alignment program (in our case, Bowtie 2 [29]) to estimate the likelihood of individual reads based on their alignment along the assembly. This approach is substantially faster than the more accurate dynamic programming algorithm that computes the cumulative likelihood of all possible alignments of a read against the assembly.

**Figure 2:**
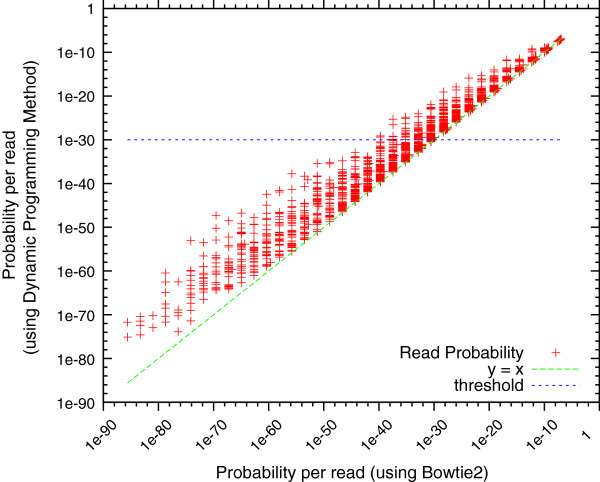
**Comparison of the read probability calculation methods for *S. aureus * with 4,788,174 reads.** Each mark on the plot represents a single read. The read’s position is determined by the probability calculated from our dynamic programming method (y-axis) and Bowtie 2 (x-axis). Points on the line *y* = *x* denote reads that were given the same probability by both methods. Since Bowtie 2 only finds the best alignment, it usually reports a slightly lower probability. A probability threshold of 1e-30 is shown for the dynamic programming method. The read probabilities that fall below this threshold would be rounded up to 1e-30 during LAP computation.

Figure 2 compares the per-read likelihood values with respect to the complete genome sequence of *Staphylococcus aureus*, using data provided by the GAGE competition. In this plot, each read is represented by a point whose coordinates represent the corresponding likelihood scores computed through full dynamic programming (y axis) and from Bowtie 2 alignments (x axis). As the full dynamic programming approach sums over all possible alignments, the corresponding likelihood values are higher (points occur above the diagonal) than those estimated by Bowtie 2. The difference between the two methods becomes less noticeable as the likelihood increases as more of the probability mass is concentrated around the best alignment of a read to the reference.

### The likelihood scores correlate with reference-based validation

The recent assembly competitions GAGE [16] and Assemblathon 1 [15] relied on a combination of *de novo* and reference-based metrics to compare and rank different assemblies. For the majority of these datasets, a complete or high-quality draft sequence was available, allowing the authors to objectively determine all the errors in the assemblies by aligning them to the reference sequences. Based on this information, the GAGE and Assemblathon 1 teams proposed several assembly quality metrics that simultaneously capture some aspects of the contiguity and correctness of an assembly. Here we compare our *de novo* likelihood score to these reference-based metrics.

Generally, the *de novo* LAP scores agree with the reference-corrected contiguity values (see  1, 2, and 3). Furthermore, the reference genome assembly (assumed to be the most correct reconstruction of the genome being analyzed) achieves the highest LAP score while the references derived from the closely-related organisms are considerably worse than all the other assemblies. In other words, the *de novo* LAP scores accurately capture the relative quality of the different assemblies.

**Table 1:** ***Rhodobacter sphaeroides* 2.4.1 assembly evaluation**

	**Contigs**				**Scaffolds**					
**Assembler**	**LAP**	**LAP**	**N50 (kb)**	**CN50 (kb)**	**LAP reads**	**LAP mates**	**N50 (kb)**	**CN50 (kb)**	**Unaligned**	**Unaligned**
	**reads**	**mates**							**reads**	**mates**
									**(fraction)**	**(fraction)**
ABySS	-20.924	-27.365	5.9	4.2	-20.929	-27.320	9	5	0.228	0.524
Allpaths-LG	**-20.795**	**-27.141**	42.5	**34.4**	**-20.796**	**-27.099**	**3,192**	**3,192**	**0.212**	**0.441**
Bambus2	-21.528	-27.439	93.2	12.8	-21.531	-27.424	2,439	2,419	0.270	0.501
CABOG	-22.550	-27.749	20.2	17.9	-22.550	-27.714	66	55	0.345	0.540
MSR-CA	-21.496	-27.407	22.1	19.1	-21.497	-27.324	2,976	2,966	0.268	0.478
SGA	-20.896	-27.575	4.5	2.9	-21.030	-27.416	51	51	0.237	0.541
SOAPdenovo	-20.816	-27.160	**131.7**	14.3	-20.816	-27.152	660	660	0.214	0.453
Velvet	-20.903	-27.314	15.7	14.5	-20.907	-27.246	353	270	0.219	0.471
*R. sphaeroides* ATCC 17025	-29.391	-29.973	3,218	3,218	-29.391	-29.973	3,218	3,218	0.813	0.904
*R. capsulatus*	-29.953	-29.997	3,739	3,739	-29.953	-29.997	3,739	3,739	0.978	0.995
*truth*	-20.769	-27.071	3,189	3,189	-20.769	-27.071	3,189	3,189	0.209	0.432

Assembly likelihood scores for *Rhodobacter sphaeroides* 2.4.1 from the GAGE project [15]. The results are presented separately for the contigs and scaffolds and include the number of unassembled reads (singletons), the LAP scores computed on unmated reads (LAP reads) or mate-pairs (LAP mates), the N50 contig/scaffold sizes (N50), and the reference-corrected N50 contig/scaffold sizes (CN50). The best (maximum) value for each genome-measure combination is highlighted in bold. The results for the reference assembly (either complete genome or high-quality draft) is given in the row marked *truth*. In addition, we provide the results for a closely related strain and species. All values, except the LAP scores, were taken from the GAGE publication. A threshold probability of 1e-30 was used for calculating the LAP scores. The standard deviations for the LAP’s reads and LAP’s mates scores are 0.00685 and 0.00969, respectively.

**Table 2:** ***Staphylococcus aureus *USA300 assembly evaluation**

	**Contigs**				**Scaffolds**					
**Assembler**	**LAP**	**LAP**	**N50 (kb)**	**CN50 (kb)**	**LAP reads**	**LAP mates**	**N50 (kb)**	**CN50 (kb)**	**Unaligned**	**Unaligned**
	**reads**	**mates**							**reads**	**mates**
									**(fraction)**	**(fraction)**
ABySS	**-16.608**	-24.692	29.2	24.8	**-16.611**	-24.584	34	28	**0.318**	0.522
Allpaths-LG	-18.018	-23.974	96.7	**66.2**	-18.018	**-23.760**	1,092	**1,092**	0.374	**0.494**
Bambus2	-18.083	-24.256	50.2	16.7	-18.085	-23.899	1,084	1,084	0.375	0.503
MSR-CA	-18.282	-24.258	59.2	48.2	-18.282	-23.926	**2,412**	1,022	0.389	0.508
SGA	-17.937	-27.019	4	4	-18.250	-24.906	208	208	0.384	0.578
SOAPdenovo	-17.830	**-23.892**	**288.2**	62.7	-17.830	-23.862	332	288	0.362	0.499
Velvet	-17.867	-24.258	48.4	41.5	-17.867	-23.925	762	126	0.363	0.503
*S. aureus* 04-02981	-19.960	-25.314	2,821	2,821	-19.960	-25.314	2,821	2,821	0.456	0.572
*S. epidermidis*	-29.635	-29.951	2,499	2,499	-29.635	-29.951	2,499	2,499	0.972	0.988
*truth*	-17.741	-23.509	2,873	2,873	-17.741	-23.509	2,873	2,873	0.358	0.473

Assembly likelihood scores for *Staphylococcus aureus* USA300 from the GAGE project [15]. The results are presented separately for the contigs and scaffolds and include the number of unassembled reads (singletons), the LAP scores computed on unmated reads (LAP reads) or mate-pairs (LAP mates), the N50 contig/scaffold sizes (N50), and the reference-corrected N50 contig/scaffold sizes (CN50). The best (maximum) value for each genome-measure combination is highlighted in bold. The results for the reference assembly (either complete genome or high-quality draft) is given in the row marked *truth*. In addition, we provide the results for a closely related strain and species. All values, except the LAP scores, were taken from the GAGE publication. A threshold probability of 1e-30 was used for calculating the LAP scores. The standard deviations for the LAP’s reads and LAP’s mates scores are 0.00740 and 0.0105, respectively.

**Table 3:** ***Homo sapiens* chr 14 assembly evaluation**

	**Contigs**				**Scaffolds**						
**Assembler**	**LAP**	**LAP**	**N50 (kb)**	**CN50 (kb)**	**LAP**	**LAP**	**N50 (kb)**	**CN50 (kb)**	**CGAL Score**	**Unaligned**	**Unaligned**
	**reads**	**mates**			**reads**	**mates**				**reads**	**mates**
										**(fraction)**	**(fraction)**
ABySS	-18.473	-23.801	2	2	-18.474	-23.787	2.1	2	-15.21×10^8^	0.257	0.504
Allpaths-LG	-15.813	-21.413	36.5	21	-15.824	-21.314	**81,647**	**4,702**	-13.11×10^8^	0.115	0.239
Bambus2	-18.606	-23.474	5.9	4.3	-18.642	-23.343	324	161	-	0.258	0.422
CABOG	**-15.625**	**-21.128**	**45.3**	**23.7**	**-15.626**	**-21.041**	393	26	**-12.25 *×*1**0^8^	0.109	**0.229**
MSR-CA	-16.421	-22.428	4.9	4.3	-16.436	-21.861	893	94	-	0.122	0.276
SGA	-15.712	-22.990	2.7	2.7	-16.909	-22.326	83	79	-	0.134	0.328
SOAPdenovo	-15.702	-21.705	14.7	7.4	-15.734	-21.594	455	214	*	**0.101**	0.269
Velvet	-18.000	-23.468	2.3	2.1	-18.140	-23.375	1,190	27	-	0.214	0.442
*truth*	-15.466	-21.001	107,349.50	107,349.50	-15.466	-21.002	107,349.50	107,349.50	-11.25 ×10^8^	0.093	0.211

Assembly likelihood scores for human chromosome 14 from the GAGE project [15] using a 10,000 read sample. The results are presented separately for the contigs and scaffolds and include the number of unassembled reads (singletons), the LAP scores computed on unmated reads (LAP reads) or mate-pairs (LAP mates), the N50 contig/scaffold sizes (N50), and the reference-corrected N50 contig/scaffold sizes (CN50). The best (maximum) value for each genome-measure combination is highlighted in bold. The results for the reference assembly (either complete genome or high-quality draft) is given in the row marked *truth*. In addition, we provide the results for a closely related strain and species. CGAL scores calculated from the long insert library were taken from the CGAL publication. The authors only provided scores for the top three assemblies (Bowtie2 could not successfully map reads to the SOAPdenovo assembly). All values, except the LAP and CGAL scores, were taken from the GAGE publication. A threshold probability of 1e-30 was used for calculating the LAP scores. The standard deviation for both the LAP’s reads and LAP’s mates scores is 0.15.

It is important to note that there are several exceptions to these general observations. In the case of *S. aureus* USA300 (Table 2), the read-based LAP scores for the Abyss assembly (computed on both contigs and scaffolds) are better than those obtained for the reference genome, contradicting our intuition, since ABySS’s reference-corrected contiguity is worse. This result highlights the importance of accurately modeling the sequencing experiment when computing the LAP scores. Once mate-pair information is taken into account, the LAP scores correctly identify the best assembly. This phenomenon is due to the fact that the Abyss assembly is able to incorporate more of the reads however their placement in the assembly is inconsistent with the mate-pair linkage information.

In the case of the human chromosome 14 assembly (Table 3), the scaffold-based results do not agree with the reference-corrected contiguity values: the CABOG assembler outperforms Allpaths-LG in all but the corrected scaffold N50 measure. This result highlights the inherent difficulty of assessing the assembly quality even when a reference sequence is available. In this case, Allpaths-LG scaffold covers a larger stretch of the genome; however, at the cost of errors both within the contigs and in their relative placement. Furthermore, the CABOG assembler is able to align nearly 0.1% more mate-pairs than Allpaths-LG, despite having a far smaller scaffold size.

The Assemblathon 1 competition [15] further demonstrated the difficulty of accurately assessing the relative quality of genome assemblies even when a correct reference sequence is available. The authors developed a collection of quality metrics that measure the stretch of a correctly assembled sequence (for example, contig path NG50 and scaffold path NG50), the amount of structural errors (such as insertions, deletions, and translocation), the long range contiguity (for example, the average distance between correctly paired genomic loci), the number of copy number errors, and the coverage within the assembly or only within coding regions. All these metrics were computed with respect to two reference haplotypes, from which the read data were simulated. The authors ranked the different assemblies by each of the metrics and used the combined information to rank the assemblies quality.

**Figure 3:**
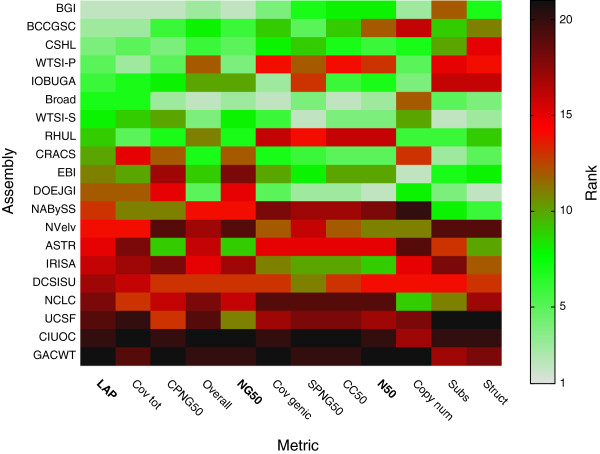
**Comparison between LAP scores and the rankings of the top assemblies generated in the Assemblathon 1 competition.** The colors represent the relative ranking provided by the individual metrics (best - green, worst - red): log average probability (LAP), overall coverage (Cov tot), contig path NG50 (CPNG50), sum of all rankings from Assemblathon 1 (Overall), weighted median contig size based on estimated genome size (NG50), coverage within coding sequences (Cov genic), scaffold path NG50 (SPNG50), length for which half of any two valid columns in the assembly are correct in order and orientation (CC50), weighted median contig size based on total assembly size (N50), proportion of columns with a copy number error (Copy num), total substitution errors per correct bit (Subs), and sum of structural errors (Struct). Column descriptions and underlying data obtained from Table 3 in Earl et al. [15]. Columns are sorted according to the level of concordance with the LAP ranking. *De novo* measures are highlighted in bold.

In Figure 3, we compare the rankings provided by our LAP score to the rankings generated by the Assemblathon 1 competition. In addition to LAP, the figure also includes two variants of the most commonly used *de novo* measure of assembly size, N50 – the weighted median contig size, that is, the length of largest contig *c* such that the total size of the contigs larger than *c* exceeds half of the genome size. N50 uses the total assembly size as a proxy for the genome size while the NG50 value uses a guess of the actual genome size to compute the N50 value. The more accurate estimation of the genome size results in a better NG50’s ranking, confirmed by the concordance with our LAP score.

The overall coverage measure (percentage of the reference haplotypes covered by a particular assembly) correlates better with the LAP score than the other metrics. This result is not surprising as the LAP score is strongly affected by the number of the reads that can be correctly mapped to an assembly, which is ultimately correlated with the concordance between the assembly and the correct reference sequence. Interestingly, the overall rankings differ between LAP and the conclusions of the Assemblathon 1 study. Our analysis suggests that the BGI assembly is the best while the Assemblathon 1 picked the Broad assembly as the winner. This discrepancy can be partially explained in part by the Broad’s high performance within the genic regions (LAP does not distinguish between genic and inter-genic segments) and the large weight placed on the BGI’s assembly’s poor performance in terms of substitution errors which have a relatively small effect on the LAP score.

It is important to note that while LAP and the Assemblathon 1 results disagree in the exact total ranking of the assemblies, the top 11 assemblies are the same, meaning they are fundamentally of better quality than the remaining 9 assemblies presented in the Assemblathon 1 paper. In fact, the Assemblathon overall score jumps from 74 for the 11th (WTSI-P) assembly to 99 for the 12th (DCSISU) assembly, indicating a substantial qualitative difference. This is also reflected in the corresponding jump in the LAP score from -37.326 to -39.441 for the 11th (DOEJGI) and 12th (NABySS) assemblies, respectively.

### The effect of a contaminant DNA on the assessment of the assembly quality

The Assemblathon 1 dataset provides an interesting challenge to the assembly assessment. The simulated libraries, generated in this project from the human chromosome 13, also included approximately 5% of the contaminant DNA from an *Escherichia coli* genome to simulate commonly encountered laboratory contamination that possibly occur due to the fragments of the cloning vector being sequenced along with the genome of interest. The participants to the Assemblathon 1 competition were given the option to either remove the contaminant DNA prior to assembly or retain the corresponding sequences in their assembly. This decision has little effect on comparison between the resulting assembly and the correct reference genome in the Assemblathon 1; however, the ability of an assembler to correctly reconstruct the contaminant genome significantly affects the corresponding LAP score.

**Figure 4:**
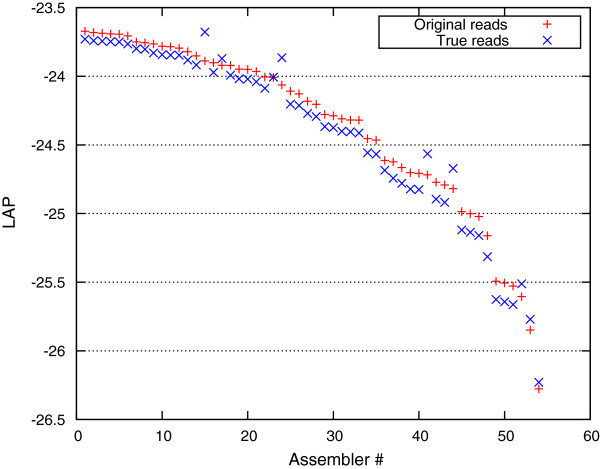
**Effect of a contaminant DNA on the computation of the LAP scores.** Red crosses are the LAP scores computed on the entire read set (including contamination). Blue crosses are the LAP scores computed only on the ‘true’ reads that map to the genome of interest. The corresponding LAP scores are quite similar (those obtained from a smaller set of reads are correspondingly smaller) except for those of assemblies that removed the contaminant DNA prior to assembly, and receive a boost in the LAP scores obtained on the “true” data.

Indeed, the LAP score (Figure 4) computed from the entire set of reads (the red crosses) and that computed after the contaminant reads were removed (the blue crosses) are strongly correlated, the latter scores are slightly lower since they were computed on the smaller dataset. In several cases, the assembly was performed after removal of the contaminant DNA (see “jumps” in Figure 4). These assemblies are penalized by our framework for not assembling the contaminant DNA, a penalty that is removed once the same set of reads is used for both assembly and quality assessment.

It is important to stress that the LAP scores can only be meaningfully compared across the assemblies generated from the same read set. If a contaminant is known it should either be removed from or retained within the dataset for all assemblers being compared; otherwise, the corresponding scores can not be directly compared. Note that this property is not unique to our measure: ignoring or assembling contaminant DNA also affects other traditional measures of quality, such as the N50 value or any reference-based measures, for example, in the case where the contaminant DNA shares significant similarity to the genome being assembled.

In practice, a ‘contaminant’ is not known *a priori*, and its definition depends on the specifics of an experiment. In general, it is difficult, if not impossible, to distinguish between environmental contaminants and true artifacts in the data, both in the context of metagenomic projects and in the case of isolate genomes. For example, the *Bacillus anthracis* samples from the bioterror attack in 2001, which were originally presumed to be uniform, contained a mixture of very closely related strains, and the characteristics of this mixture formed an important forensic marker in the investigation [31].

### A useful application: tuning assembly parameters

**Figure 5:**
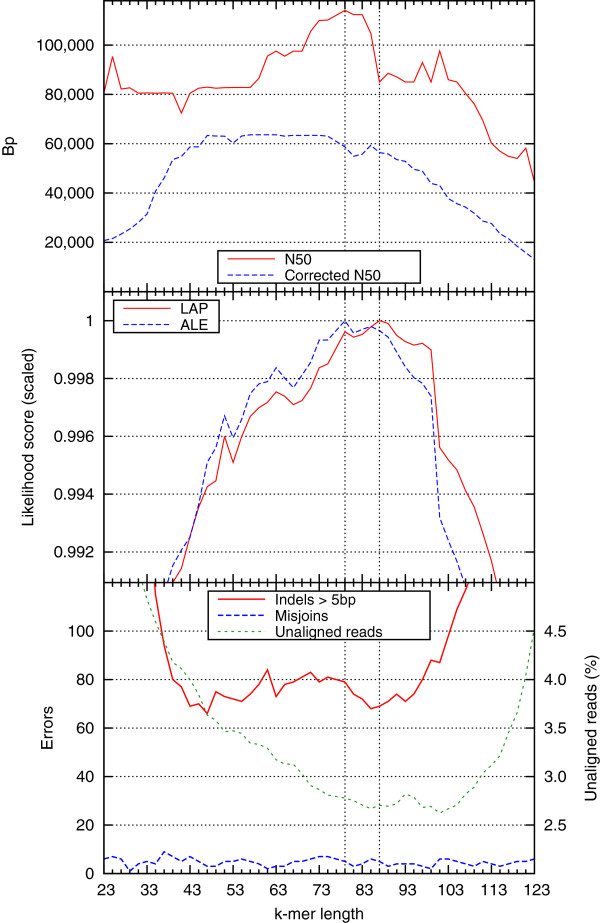
**Tuning SOAPdenovo k-mer parameter using LAP.** LAP, N50, and corrected N50 are plotted for various SOAPdenovo assemblies of *E. coli* K12 MG1655 dataset for different *k*-mer sizes (k = 23-123). ALE [27] scores are plotted alongside the LAP to show the differences between their underlying likelihood models. Also included is a breakdown of the errors along with the percentage of the unaligned reads for the various SOAPdenovo assemblies. Two vertical lines (at k = 79 and k = 87) correspond to the maximum ALE and LAP score, respectively.

Our discussion so far has focused on comparing the output of different assembly software with the goal of choosing the best assembler for a particular dataset. The developed probabilistic framework can also be used to better choose the combination of parameters that allow a particular assembly to achieve better results. To demonstrate this use case, we target the task of selecting the “best” (in terms of final assembly quality) *k*-mer length for a de Bruijn graph-based assembler. We focus here on SOAPdenovo assemblies of the *Escherichia coli* K12 MG1655 genome (Figure 5).

Without the availability of a reference sequence, users of assembly software usually rely on the N50 value as a proxy for the assembly quality. In this case, there is a clearly defined peak in N50 at k = 79 (114,112 bp). After adjusting for the assembly errors, there is a collection of the assemblies (k = 47-51, 55-75) with nearly identical corrected N50s (∼64,000 bp). These assemblies range in N50 from ∼80-115 kbp. Our *de novo* measure LAP shows a clear peak at k = 87, which corresponds to a corrected N50 of 59,352 bp. It is important to note that despite roughly a 7% difference from the peak in corrected N50 (k = 63), the best LAP assembly contains 4 fewer indels larger than 5 bp, while also aligns roughly 54,000 more reads.

Alongside our LAP, we plot the likelihoods calculated from another assembly evaluator framework, ALE [27]. The assembly with the highest ALE score (k = 79) corresponds to the N50 peak. Compared to the LAP selected assembly, the ALE selected assembly contains 10 more indels larger than 5 bp and has a 49% drop from N50 to corrected N50 compared to the 35% drop between those values for the LAP’s selected assembly.

## Discussion and conclusions

In this paper, we have proposed a paradigm for the *de novo* evaluation of genome assemblies. While the general paradigm could, in principle, be used to provide an objective score of assembly quality, our practical implementation of this paradigm, called the Log Average Probability (LAP), is dataset specific and should only be used to provide relative rankings of different assemblies of the same dataset. Unlike traditional measures of assembly contiguity (such as the N50 value), our reference-independent LAP scores correlate with reference-based measures of assembly quality.

We would like to stress that *de novo* measures of assembly quality, such as ours, are critically needed by researchers targeting an assembly of yet unknown genomes. The specific characteristics of the data being assembled have a significant impact on the performance of genome assemblers (in the Assemblathon 1 [15] and GAGE [16] competitions, for example, different assemblers ‘won’ the competition depending on the analyzed dataset); thus, the reference-based quality assessments cannot be reliably generalized to new genome projects.

In our paper, we have made a number of simplifying assumptions for modeling the sequencing process; specifically, that the sequencing process is uniform (both in the coverage, and the error profile), and that the reads are independently sampled from the genome (with the exception of the dependence imposed by mate-pair experiments). While our approach can detect copy number differences (unless the entire genome is exactly duplicated), it is with the caveat that sequencing biases within repetitive regions can possibly mask mis-assemblies. More precise models of the sequencing process that relax these assumptions can be easily incorporated into our framework (e.g., effects of G/C content on sequencing depth, or technology-specific error profiles). We plan to create technology-specific variants of our score to keep up with the rapid changes in the characteristics of the sequencing data as new instruments and/or chemistries become available. Furthermore, the probabilistic framework presented here can be used to incorporate other types of information on the assembly quality, for example, optical mapping data [17].

In our assembler parameter-tuning experiment, we generated assemblies of *Escherichia coli* K12 MG1655 using every allowed k-mer value. While this approach may be computationally feasible for smaller genomes, it is inefficient for very large, complex genomes. One solution would be to use an optimization strategy for selecting potential k-mer values, e.g., with simulated annealing.

While there are differences between the LAP score and recent likelihood-based metrics, ALE and CGAL, these differences are quite small (Table 3 and Figure 5). Thus, it is important to discuss the technical improvements over ALE and CGAL. ALE’s score did not perform quite as well as our LAP score on the parameter tuning experiment, and CGAL is unable to evaluate all of the GAGE assemblies due to the technical limitations of Bowtie 2. Bowtie 2 was not designed for reporting *all* read alignments, which makes it very slow on large genomes. This problem will become more prevalent as sequencing costs continue to decrease, allowing for more complex genomes to be sequenced and assembled. Our framework overcomes CGAL’s limitations by allowing users to calculate the LAP score via the dynamic programming method on a subset of the reads or by using the SAM file produced from a read alignment tool designed for finding all alignments (e.g., mrsFAST [32]).

Our original goal was not to detect assembly errors, but to provide a global measure of how good an assembly may be. We plan to extend our framework to detect assembly errors by adopting a similar approach to that demonstrated by ALE.

It is important to note that we have focused on a very specific use case for assembly – the complete reconstruction of a given genome. Assembly algorithms are used in a number of other biological applications, whose specific characteristics affect the validation of the resulting assembly. For example, studies targeting the genic regions of an organism may tolerate large-scale rearrangements as long as the individual genes are correctly reconstructed. In this context, the validation framework would penalize substitution errors and small insertions or deletions (which potentially affect gene structure) more than mis-joins within intergenic regions. Such application specific tuning is possible within the proposed overall framework, and we envisage the creation of a collection of community-supported modules that compute application-specific LAP scores.

Our discussion has focused on the assembly of single genomes, however the LAP score, as described, can also be directly used in the context of diploid genomes or metagenomic mixtures. In this case, our score implicitly assumes that the goal of the assembler is to correctly reassemble both the sequence and the relative abundances of the individual haplotypes. Assume, for example, a simple metagenomic sample that contains two organisms; one that is twice as abundant as the other one. An assembler that produces three sequences, corresponding to the three ‘haplotypes’ in the sample (whether explicitly outputting two, perhaps identical, versions of the abundant organism or reporting the copy-number difference in some other way) would obtain a better LAP score than an assembler that only reported two sequences without any indication of their relative abundance. As a result, the majority of metagenomic assemblers available today, which only output the consensus sequence and not the relative abundance of the contigs, would score poorly under our score. We hope that our work will inspire the developers of future metagenomic assemblers to also output information on the relative abundance of the reconstructed sequences, information that is critical to the analysis of the data, yet rarely reported by existing tools.

Finally, we propose that measures such as ours, which objectively capture the fit between the data being assembled and the output produced by the assembler without relying on curated reference data sets, become a standard tool in evaluating and comparing assembly tools, allowing the community to move beyond simplistic measures of contiguity such as the ubiquitous N50 measure.

## Competing interests

The authors declare that they have no competing interests.

## Authors’ contributions

MG and CH developed the LAP software. CH carried out the GAGE and Assemblathon1 experiments. CH and DS performed the assembler parameter tuning experiments. MG, CH, IA, and HL developed the underlying statistical theory. MP conceived of the study, and participated in its design and coordination and helped to draft the manuscript. All authors read and approved the final manuscript.

## Supplementary Material

Additional file 1 LAP framework software outline. Click here for file
