# Supplementary material for: De novo likelihood-based measures for comparing genome assemblies
Source: BMC Res Notes. 2013 Aug 22;6:334. doi: 10.1186/1756-0500-6-334 (PMC3765854; doi:10.1186/1756-0500-6-334)
Supplement: Additional file 1 — LAP framework software outline. [file 1756-0500-6-334-S1.pdf]

De novo likelihood-based measures for assembly validation

1 Supplementary Material

1.1 Program versions and parameters

Below we outline the specific details of all programs used in our paper and their exact parameters.

Executables and scripts for calculating the LAP are available to download from `http://assembly-eval.sourceforge.net`. Our framework is split up into the two categories, which contains the executables and scripts for computing the LAP using either alignment tools (**aligner**) or the dynamic programing methods (**dynamic**):

- **aligner**
  - calc_prob.py - calculates read probability with Bowtie 2
  - sum_prob.py - calculates LAP from read probabilities
  - gen_rand_samp.py - sample reads
- **dynamic**
  - mprobability - calculates read probability using dynamic programming method
  - mean - calculates LAP from read probabilities
  - sample - samples reads
  - stitch - stitch mate pairs together

1.1.1 Calculating LAP using the aligner tool

For the two bacterial genomes in the GAGE dataset, we calculate the LAP for single and mated reads using *all* of the reads with our aligner method: `calc_prob.py`. `calc_prob.py` is a python script that calculates the read probabilities using Bowtie 2 (version 2.0.2) with the −**very-sensitive** preset. The location of this version of Bowtie 2 must be stored in the shell variable `$BT2_HOME`.

Here is an example of how to calculate the fragment and shortjump mate pair probabilities of the *Rhodobacter sphaeroides* dataset using 5 threads with insert sizes of 180bp and 3500bp, respectively.

```
./calc_prob.py -p 5 \
    -a reads/gage/Rhodobacter_sphaeroides/Assembly/Allpaths-LG/genome.ctg.fasta \
    -1 reads/gage/Rhodobacter_sphaeroides/Data/original/frag_1.fastq,\
    reads/gage/Rhodobacter_sphaeroides/Data/original/shortjump_1.fastq \
    -2 reads/gage/Rhodobacter_sphaeroides/Data/original/frag_2.fastq,\
    reads/gage/Rhodobacter_sphaeroides/Data/original/shortjump_2.fastq \
    -X 500,4250 -I 0,3000 -o fr,rf -m 180,3500 -t 18,350 > rhodo_allpaths.prob
```

, where `-X` and `-I` are the upper and lower bounds that Bowtie2 will consider a valid mate pair alignment, respectively.

The output read probabilities are piped into the python script `sum_prob.py`, which takes in a list of read probabilities, to calculate the LAP using a threshold probability for unaligned reads:

```
cat rhodo_allpaths.prob | ./sum_prob.py -t "1e-30"
```

We also provide the script `run_calc_gage_probs.sh` to allow users to calculate the single and mate pair LAPs for the GAGE assemblies. For mate pair probability calculations we used the insert sizes provided by GAGE, with a 10% standard deviation. Further instructions are included in a README file.

The human chromosome contains approximately 15x as many reads as the bacterial sequences, so we calculate the LAP using a 10,000 read sample with the aligner method. `gen_random_sample.py` is a python script to randomly sample reads from a given list of sequence files:

```
/gen_rand_samp.py \
    -1 reads/gage/Hg_chr14/Data/original/frag_1.fastq \
    -2 reads/gage/Hg_chr14/Data/original/frag_2.fastq \
    -k 10000 \
    -o reads/gage/Hg_chr14/Data/subsets/
```

1.1.2 Calculating LAP using the dynamic programming method

For the Assemblathon 1 results we compute the LAP for each assembly using 200,000 sampled reads from the 300 and 3,000bp insert size libraries. Bowtie2 is unable to align a few reads to all locations in the Assemblathon 1 assemblies, so we use our dynamic programming method for computing the LAP. We encountered a similar issue with Bowtie 2 and the Bumble bee data from GAGE, but since GAGE did not have a reference for the Bumble bee, we did not include it in our analysis.

Here is an example of how to `sample` 200,000 of the reads of 300bp insert library, calculate the probabilities (`mprobability`), and finally, calculate the LAP (`mean`):

```
cat speciesA_300i_40x.1.fastq | sample -n 200000 | \
mprobability -a reference.fasta --print-headers | \
mean -t "1e-80"
```

Like the **aligner** method, we provide a script (`run_calc_asm1_prob.sh`) to calculate the LAP for all Assemblathon 1 assemblies.
